# Parents regulate arousal while sharing experiences with their child: a study of pupil diameter change responses

**DOI:** 10.3389/fnhum.2023.1177687

**Published:** 2023-06-16

**Authors:** Jessica Yarmolovsky, Maya Sabag, Or Lipschits, Ronny Geva

**Affiliations:** ^1^The Developmental Neuropsychology Laboratory, The Department of Psychology, Bar-Ilan University, Ramat Gan, Israel; ^2^The Susan Gonda Brain Research Center, Bar-Ilan University, Ramat Gan, Israel

**Keywords:** self-regulation, sharing, parent-child, dyadic interaction, pupil diameter change

## Abstract

**Introduction:**

Parents provide their children with their first exposures to reciprocal shared experiences, and parental modeling of socio-emotional behaviors and regulatory responses largely influences their child’s behavioral and neurological development. Some parental reactions are conscious, while others are non-volitional. This project aimed to explore parent-child pupil dilation change responses during shared interactions, specifically, whether parents’ neuro-regulatory responses when sharing experiences with their child are different than responses of children interacting with their parents or children and adult peers sharing with each other.

**Methods:**

To test this, four distinct interacting groups were recruited: (1) Parents sharing with their child; (2) Children sharing with their parent; (3) Children sharing with peers; and (4) Adults sharing with peers. All dyads engaged in a computerized shared imagery task, which facilitates communication and mental imagery during a shared experience. During the task, pupil diameter change was recorded as a measure of regulatory response.

**Results:**

Findings highlight that parents sharing with their child have lower pupil diameter change than children sharing with their parents (*p* < 0.01), children sharing with peers (*p* < 0.01), and adults sharing with peers (*p* < 0.05), While no differences were seen between children sharing with parents, children sharing with peers or adults sharing with peers.

**Discussion:**

Findings deepen the understanding of the neuroscience of parenting, by suggesting that parents, even of older children and adolescents, tend to regulate their arousal when interacting with their child, a response that proves to be unique compared to other dyad types for sharing experiences. Considering this dynamic, findings may direct future parent-led intervention methods to improve the child’s socio-emotional development.

## 1. Introduction

Parents play an essential role in their child’s development and wellbeing, providing a model for adaptive social interactions and self-regulatory abilities (Tan et al., 2020; Ratliff et al., 2022). From the first days of life, babies rely entirely on their primary caregiver for emotion regulation and support. This dependency is needed, for example, in parental calming or soothing a crying baby and responding to the biological needs that are causing the child stress (Frankel et al., 2012). To facilitate this, often the parent modulates their behavior or reaction to adapt to their child’s needs (Verde-Cagiao et al., 2022), such as adjusting movement (Brand et al., 2002), or language (Shruti et al., 2018) to accommodate the child better. As children grow and become more autonomous, the child is gradually capable of more self-regulation capacities, with the parent’s input typically remaining a crucial factor throughout the child’s development into adulthood (Morris et al., 2017a).

### 1.1. Shared experiences

One of the more common social interactions between parents and their children is sharing experiences, a setting that is thought to contribute to adaptive social development (McAuley et al., 2012). Parents provide their children with their first exposure to reciprocal shared experiences, and parental modeling of socio-emotional behaviors and regulatory responses largely influences their child’s behavioral and neurological development (Eisenberg et al., 1998; Morris et al., 2017a; Davis et al., 2018; Tan et al., 2020; Ratliff et al., 2022). Spending time together and sharing experiences with others leads to a greater sense of wellbeing (Hudson et al., 2020). Shared activities and experiences provide a base for children to learn how to interact with others competently and negotiate social relationships (McAuley et al., 2012). Parents report spending time with their children as one of their more enjoyable activities (Guryan et al., 2008). However, we know little about the neurobiological processes involved in parent-child sharing experiences. What happens in the parent-child brain during these meaningful shared interactions? Plausibly individual neural network adaptive responses will be seen for each partner. Do these responses occur the same way in the child’s and the parent’s brains? This project aimed to explore these questions, focusing on the arousal regulation of parents and their children compared to peer-interacting dyads during a shared experience task.

On a day-to-day basis, one of the most common activities that we encounter is shared experiences. That is, we look at or perform an activity together with another. By doing so, we thereby facilitate discussion or sharing with the other. Research shows that shared experiences, both positive and negative, amplify how one interprets that experience (Boothby et al., 2014). One study, for example, shows that participants experienced the same chocolate as more enjoyable when eating it together with a partner rather than alone while their partner did something else (Boothby et al., 2016). An explanation for this is that shared experiences affect arousal, as the autonomic nervous system adjusts based on social cues from the partner (Waters et al., 2014; Noy et al., 2015). Accommodation of arousal via regulation affects the other’s behavior and regulatory state (Ferrer and Helm, 2013).

### 1.2. Neuro-cognitive components of shared experiences

On a cognitive level, sharing an experience requires a theory of mind to adapt the mode, content, and speed in which you share (Wu and Su, 2014). Doing so successfully allows one to maximize the level of sharing so that the partner receives the desired information. Neuroimaging research gives us a clue into the inter-relationship of brain-behavior responses across the parent-child dyad. For example, positive maternal behaviors are prospectively related to the development of neural structures associated with emotional reactivity and regulation in the child (Whittle et al., 2014). From the parental brain perspective, increased connectivity in the empathy networks in the parent during infancy is prospectively related to a child’s ability to engage in more regulation strategies (Abraham et al., 2016). In children and adolescents, the neurobiological processes involved in regulation continue to develop, and as such, they rely heavily on their parents to direct them (Morris et al., 2017b).

### 1.3. The parent-child shared experience

Dyadic interactions generally imply bi-directional and mostly equal contributions, often represented by synchrony (Harrist and Waugh, 2002). However, this may not always be the case in the parent-child dyad, as each member has different interests and goals. The parental role involves caretaking, sharing resources, and teaching/modeling for their child (Thai et al., 2019). This role is especially complex in the understudied age group of older childhood, as this is a time in which children begin to gain a strong sense of agency and start to experience the world independently for the first time. On the other hand, they are still very reliant on their parents and very much affected by positive interactions and reactions from their caretakers (Cirelli et al., 2014). Some parental reactions are conscious, such as modeling a motor response or providing instructions, while other parental reactions are non-volitional. These latter ones were of interest in the current study.

Given the effects of shared experiences on arousal and autonomic nervous system adjustment between partners (Waters et al., 2014; Noy et al., 2015), we aimed to explore individual autonomic nervous system responses during shared interactions. The literature suggests that the mother’s stress-induced autonomic nervous system reactivity is passed on to their child, who was not directly exposed to the stressor (Burkhouse et al., 2014; Waters et al., 2014). For example, children of depressed mothers were found to have increased arousal, measured by pupil diameter, toward sad emotional stimuli (Burkhouse et al., 2014). This idea of stress contagion would plausibly be seen in the reverse direction as well, such that more arousal-regulated autonomic nervous system reactivity from the mother would lead to feelings of regulation in the child. Given this notion, it is plausible that parents will adjust or manipulate their arousal regulation when interacting with their child to minimize the potential effects of stress contagion, an effect that is not expected while children interact with their parents or during peer-shared experiences.

### 1.4. Pupil dilation in shared experiences

One effective autonomic nervous system reactivity indicator is pupil dilation (Wang et al., 2018; Maier and Grueschow, 2021). Beyond considering the reflexive light response, pupillary responses have been known to increase when experiencing emotionally arousing stimuli and decrease during times of low arousal, representing sympathetic nervous system reactivity (Bradley et al., 2008). Pupil constriction and dilation have been correlated with neural activity in regions responsible for emotion regulation, such as the dorsolateral prefrontal cortex (Siegle et al., 2003) or anterior cingulate cortex (Critchley et al., 2005) as well as with locus coeruleus—norepinephrine system activation (Laeng et al., 2012). This activation type represents the arousal system and offers a measure of cognitive and emotional regulation (Kinner et al., 2017; Mathôt, 2018; Grueschow et al., 2020; Maier and Grueschow, 2021). The use of pupillometry to measure pupil diameter employs a non-invasive means for capturing unbiased arousal responses during live interactions, making it an ideal measure for live shared experiences.

Therefore, the current study measured real-time pupillary responses in parents when they share with their child, children when they share with their parent, as well as adults and children sharing with peers to explore the arousal behaviors of the individual. We hypothesized that parents interacting with their child would present more arousal regulation during their active role during a task that involves a high level of sharing, evidenced by lower pupil diameter, compared to their child, as well as compared with adults interacting with age-matched peers. On the other hand, due to their independent contribution to the parent-child dyad, children are expected to show similar pupil diameter patterns as they would when sharing an experience with another child.

## 2. Materials and methods

The current research recruited four distinct interaction groups to enable comparisons of parents sharing with their child, children sharing with their parents, children sharing with peers, and adults sharing with peers. Pupil diameter was recorded for all interacting individuals to measure arousal regulation during a live natural, shared experience. The unique paradigm allowed real-time measures of regulatory responses while sharing, focusing on individuals’ pupillary responses while describing what they see to their partners.

### 2.1. Participants

Seventy-four participants in one of 4 interaction mode conditions took part in the study: (a) 22 adults sharing an experience with peer strangers (11 dyads; mean age = 21.7 years, SE = 1.06; 73% female); (b) 24 children sharing an experience with peer strangers (12 dyads; mean age = 12.3 years, SE = 0.61; 22% female), (c) 12 children sharing an experience with their parents (12 dyads; mean age = 9.45 years, SE = 0.36; 58% female), and (d) 12 parents sharing an experience with their children (12 dyads; mean age = 45.3 years, SE = 1.32; 91% female). Note that children sharing an experience with their parents are the partners of the parents sharing an experience with their children. One child from the child-peer group and one parent from the parent-child group were excluded due to a computer malfunction that resulted in no gaze data file production. An additional child from the child-peer group was excluded from the analysis due to too much missing gaze data. The children’s age group was chosen because it represents a period of relative autonomy and increased interest in social networking, though there is still an essential reliance on parental support (McElhaney et al., 2009). Further, neuropsychological development tends to stabilize around this period (Korkman et al., 2001). The adult peer sample consisted mainly of university students, as this is a time and environment in adulthood in which it is reasonably natural to interact with peer strangers who share everyday experiences. All peer dyads were matched for age and gender. Pairs consisted of individuals who did not know each other but came from a cohort who naturally are likely to interact in classes or extracurricular activities. Some pairings recognized the other from class or peer environments, but no pairs defined each other as close friends. By choosing individuals who do not know each other but are likely to interact in a natural setting, we promoted a comfortable social interaction without introducing confounding effects of heterogeneous social abilities and friendship dynamics (Becht et al., 2020).

We let the family decide whether the mother or father would participate in the research to preserve an inclusive policy and not infringe on natural family specific dynamics. This led to only one father participating in the parent-child group.

### 2.2. Experimental procedures

#### 2.2.1. The shared imagery task (SIT)

**FIGURE 1:**
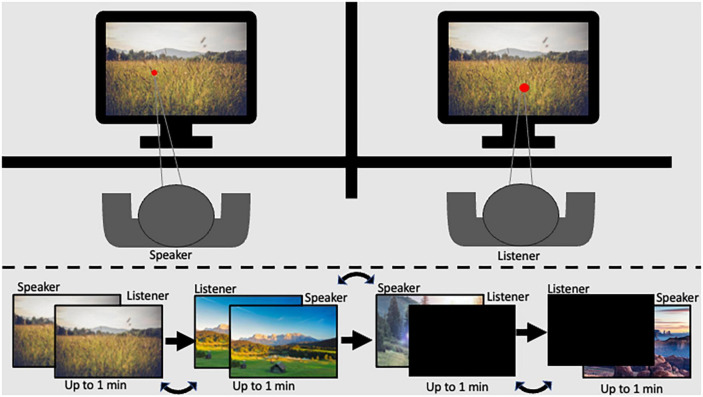
Graphical scheme of the experimental setup legend: **(top)** panel represents the participant setup in the experimental room; the **(bottom)** panel represents the order of stimulus presentation.

The Shared Imagery Task (SIT), designed for the current research aims, facilitates communication and mental imagery during a shared experience. The task allows participants to experience a stimulus together and communicate that experience to a partner while measuring pupil diameter. In the current study, participants were seated one next to the other, each 60 cm from their respective eye tracker. A barrier was placed between the participants in a way that did not allow partners to see the other’s monitor but did allow dyads to see each other and feel the presence of their partner (Figure 1).

The SIT consists of two levels of shared experience with active speaking roles: (1) High level: Both participants in the dyad viewed the same image on their respective monitors while one participant described to the other what they saw. (2) Medium level: One participant viewed and described a stimulus on their monitor while their partner viewed a black screen. Each level was repeated twice so that both dyad members had a turn as the speaker. Speaker and listener order was assigned at random. Additionally, the two levels of shared experience were presented in counterbalanced order. Images covered the entire monitor, and participants were given up to 1 min to describe the image. Stimuli remained displayed on the screen for the full minute or until participants indicated they had finished describing the image. The luminance level in the experimental room was 32 lux. To minimize the effect of luminance, images with consistent pixel intensity levels were chosen (mean intensity ranging from 101.46 to 117.85; and SD 64.75–79.43), resulting in illumination of 24–31 lux at a distance of 60 cm from the screen (measured using Hydrolux LX1010BS lux meter). Further, data cleaning and baseline procedures were employed to minimize noise, as described in the methods section (Carter and Luke, 2020).

#### 2.2.2. Individual finger tapping task

This task acted as a baseline measure of pupil diameter change when participants performed a task on their own. Participants were instructed to press the space bar as many times as possible while a cross was presented on the monitor and to stop pressing when the cross disappeared. The cross appeared for 5000 ms with a break of 2000 ms. The task ended once the participant reached three consecutive pressing trials for which the difference in the number of presses was less than five or up to 6 trials.

### 2.3. Experimental measures

#### 2.3.1. Dual eye tracking protocol

Each participant in the dyad viewed a duplicated version of the SIT task on their monitor equipped with a Tobii TX-300 or a Tobii X2-60 eye tracker. Both eye-tracking systems employ binocular eye tracking through near-infra-red diodes to generate reflection on the corneas of the user’s eyes. The systems tracked both eyes to a rated accuracy of 0.5° and sampled at 60 Hz. Each participant first underwent a successful 5-point calibration on their respective eye tracker before beginning the experimental task. The Tobii TX-300 and X2-60 were connected via LAN connection using a two-computer setup, with one computer running E-prime software to record the X2-60 tracker data; and the other running Tobii Studio software, used to record TX-300 data. Output files from Eprime and Tobii Studio for each dyad member were adjusted to allow participant comparison.

#### 2.3.2. Pupil diameter regulatory reactivity curve calculations

**FIGURE 2:**
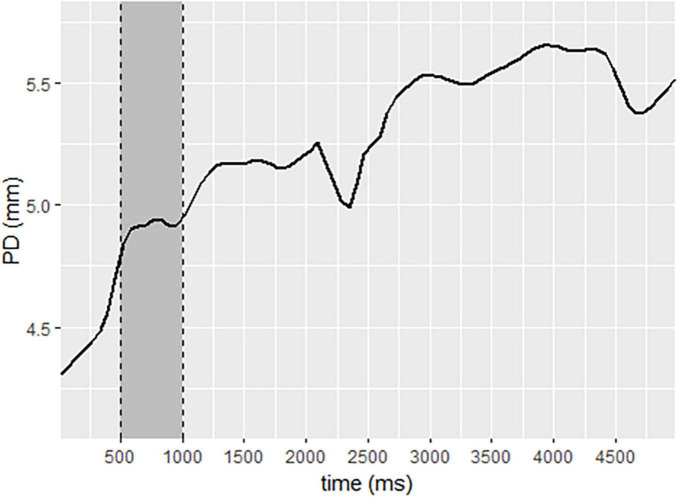
Pupil diameter change sample, demonstrating baseline selection to neutralize the initial light response. Legend: data selected for baseline is represented in the gray area. The solid line represents pupil diameter change.

Pupil diameter change (ΔPD) response was calculated per participant per trial using the Pupillometry R package protocol (Forbes, 2020). R Studio (R Development Core Team, 2010). First, the left and right pupil data were regressed against the other and averaged. Next, the data were down-sampled from 60 to 10 Hz (Siegle et al., 2004). Trials missing more than 75% of data were excluded from analysis (26 trials), and participants missing more than 75% of their total data were excluded (1 participant). Data were filtered using a median filter with a rolling window of 11 degrees. Blinks were removed and linearly interpolated. Baseline correction was applied by subtracting individual baselines from the pupil data, yielding a measure of ΔPD. The baseline pupil size was calculated as the mean pupil size during 500 ms of each trial that occurred in the second half of the first second after the initial light response was neutralized (Mathôt et al., 2018), as demonstrated in Figure 2. Pupil diameter data for each participant was calculated per experimental block of the SIT task.

#### 2.3.3. Determination of windows of significance

Differences in pupil diameter change between individuals in each interaction group were compared by employing consecutive one-tailed *t*-tests at each time point along the PDΔ waveform (Siegle et al., 2008) between parents and their children, children and their parents, children interacting with peers, and adults interacting with peers. To control for high levels of autocorrelation that occur between pupil dilation waveforms, Guthrie and Buchwald’s (1991) technique was implemented, using Monte Carlo simulations, to define the region over which continuous points of significant one-tailed *t*-tests along the waveform could be considered significant (Siegle et al., 2004). Using this technique, our data yielded that a 1.3-s window at *p* < 0.1 is considered significant at *p* < 0.05.

## 3. Results

### 3.1. Initial data analysis

#### 3.1.1. Gender and age differences

To ensure that gender differences did not account for the pupil diameter findings, an ANOVA was conducted across all participants, with gender as the independent variable and average pupil dilation as the dependent variable. Results show that no significant gender differences were found for average pupil diameter change during the SIT task [*F*_(1,317)_ = 0.370, *p* = 0.54].

**FIGURE 3:**
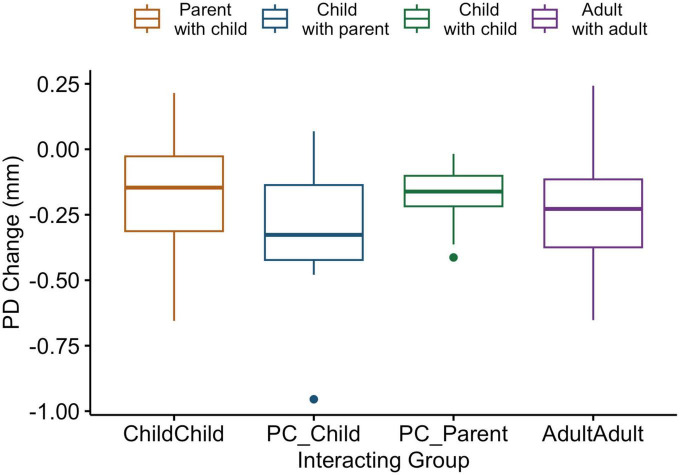
Box and whiskers plot indicating pupil diameter change as a function of group in an individual computerized task. Legend: The center line represents the median, and the outer lines of the box represent the interquartile range. The whiskers extend to 1.5 times the interquartile range.

To address discrepancies between the ages of adult peer dyads and parents in the parent-child dyads, pupil diameter data change from a computerized finger-tapping task performed individually was analyzed. ANOVA comparing pupil diameter change in all conditions indicate that parents show similar pupillary change patterns to all individuals in the other interacting groups in this individual task [*F*_(3,60)_ = 1.04, *p* = 0.38; Figure 3].

#### 3.1.2. SIT shared experience level analysis

To assess differences between the two levels of shared experiences in the SIT, a two-way ANOVA comparing pupil diameter change as a function of SIT sharing levels and interaction group was conducted. Results revealed a significant group main effect [*F*_(3,126)_ = 3.04, *p* = 0.03], an insignificant sharing level main effect [*F*_(1,126)_ = 0.10, *p* = 0.76], and no sharing level by group interaction effect [*F*_(3,126)_ = 0.52, *p* = 0.67]. *Post hoc* analysis indicates that pupil diameter changes for parents sharing with their child were smaller compared to children sharing with parents across levels (*p* = 0.02). These findings highlight that pupillary reactivity change differences between groups occur to the same degree irrespective of experience level. Given the lack of significant differences between levels, the speaking role during the high-level shared experience was analyzed for the purposes of the current study. This was chosen because it represents an especially salient case of sharing, during which both partners experience the same visual stimuli, and participants must actively share with their partner.

#### 3.1.3. Pupil diameter change comparisons between interaction modes

**FIGURE 4:**
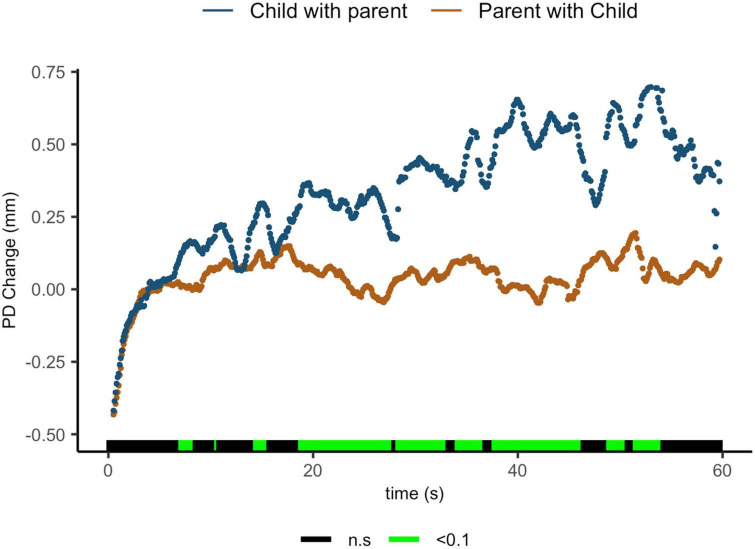
Parent with child and child with parent pupil diameter change regulatory reactivity curve comparisons. Legend: Areas of significance are noted along the *x*-axis. *P* < 0.05 is represented in green. Lines represent baseline rendered pupil diameter change.

Findings indicate that parents sharing with their children showed lower ΔPD than children sharing with their parents. Regions of significance can be seen from 7.1 to 8.4 s [t(15.6) = 2.05, *p* = 0.058, *d* = 0.88]; from 14.4 to 15.6 s [t(18.3) = 2.26, *p* = 0.036, *d* = 0.99]; from 18.8 to 27.8 s [t(18) = 3.56, *p* = 0.002, *d* = 1.59]; from 28.3 to 33.1 s [t(12.6) = 2.99, *p* = 0.011, *d* = 1.39]; from 34.1 to 36.7 s [t(7.2) = 2.62, *p* = 0.034, *d* = 1.43]; from 37.7 to 46.3 s [t(8.2) = 3.54, *p* = 0.007, *d* = 1.90]; from 48.9 to 50.6 s [t(5.4) = 2.40, *p* = 0.06, *d* = 1.42]; and from 51.5 to 54.1 s [t(15.6) = 3.2.05, *p* = 0.06, *d* = 1.63] (Figure 4).

**FIGURE 5:**
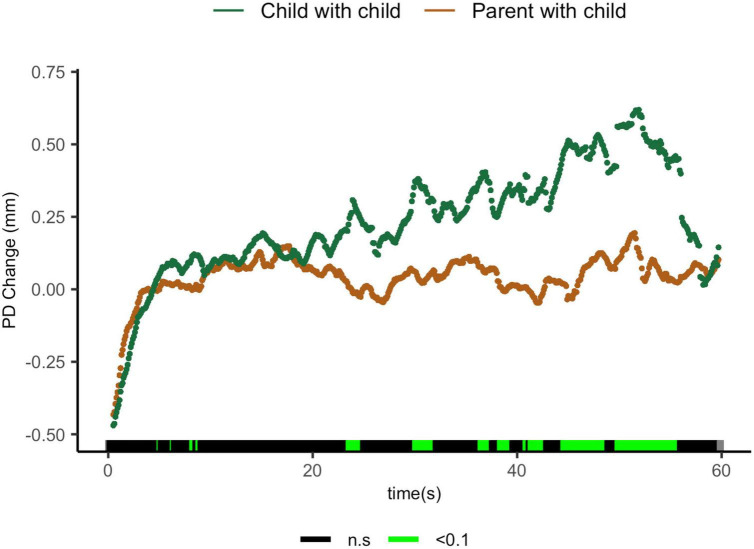
Parent with child and child-peer pupil diameter change regulatory curve comparisons. Legend: Areas of significance are noted along the *x*-axis. *P* < 0.05 is represented in green. Lines represent baseline rendered pupil diameter change.

Comparisons of parents sharing with their children and children sharing with their peers suggest that parents exhibit lower ΔPD from 23.5 to 24.6 s t(27) = 1.96, *p* = 0.06, *d* = 0.70; from 30 to 31.9 s t(17) = 2.35, *p* = 0.031, *d* = 1.02; from 41.3 to 42.7 s t(11.1) = 2.31, *p* = 0.041, *d* = 1.12; from 44.5 to 48.7 s t(9.5) = 2.34, *p* = 0.04, *d* = 1.15; and from 49.8 to 55.8 s t(8.4) = 3.00, *p* = 0.02, *d* = 1.57 (Figure 5).

**FIGURE 6:**
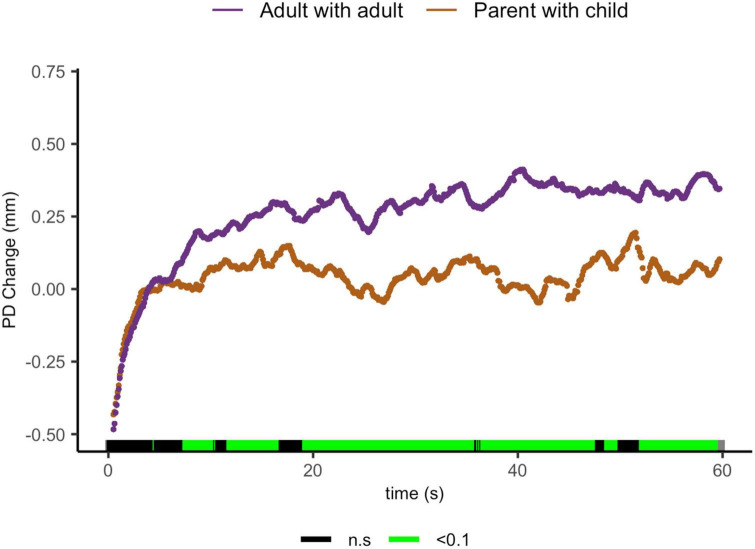
Parent with child and adult-peer pupil diameter change regulatory curve comparisons. Legend: Areas of significance are noted along the *x*-axis. *P* < 0.05 is represented in green. Lines represent baseline rendered pupil diameter change.

And finally, parents sharing with their child showed lower ΔPD than adults sharing with peers from 7.5 to 10.4 s [t(29.4) = 2.63, *p* = 0.013, *d* = 0.851], from 11.8 to 16.8 s [t(19.6) = 2.27, *p* = 0.034, *d* = 0.85]; from 19.2 to 35.9 s [t(28.7) = 2.92, *p* = 0.01, *d* = 1.00]; from 36.6 to 47.7 s [t(24.4) = 2.94, *p* = 0.007, *d* = 1.09]; from 48.7 to 49.9 s [t(23.9) = 1.99, *p* = 0.058, *d* = 0.764]; and from 51.2 to 59.7 s [t(20.2) = 2.83, *p* = 0.01, *d* = 1.15, Figure 6].

**FIGURE 7:**
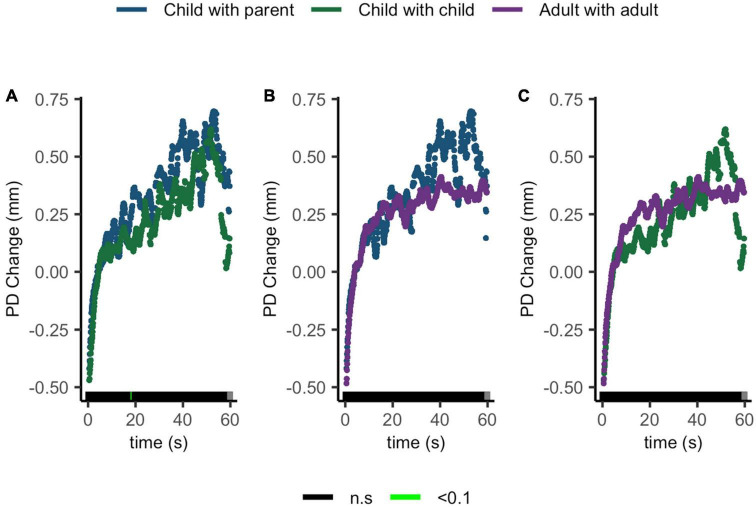
Pupil diameter change regulatory curve comparisons of **(A)** child with parent and child-peer; **(B)** child with parent and adult-peer; and **(C)** child-peer and adult-peer. Legend: Areas of significance are noted along the *x*-axis. *P* < 0.05 is represented in green. Lines represent baseline rendered pupil diameter change.

Compatible with our hypothesis, no ΔPD differences were seen between children sharing with their parents and children sharing with peers, children sharing with their parents and adults sharing with peers, or children sharing with peers and adults sharing with peers (Figures 7A–C).

Overall findings indicate that parents present with lower pupil diameter change than all other interacting groups, while other interacting partners do not differ from each other.

## 4. Discussion

The current study explored arousal responses during live interactions by measuring pupillometry among parents and their children during natural sharing and compared these behaviors to interactions between children and adults while sharing with peers. Findings highlight a unique arousal regulation profile among parents, measured by pupil diameter change, in which parents show lower arousal levels when sharing with their children compared to sharing individuals in all other groups. Given the importance of regulation abilities on the developmental outcome and the parent’s critical role in their child’s socio-emotional wellbeing (Tan et al., 2020), these findings provide important insights into the neuro-behavioral dynamics that occur during live parent-child shared experiences.

Notably, the current findings highlight that while parents exhibit lower arousal levels when sharing with their children than others sharing with peers, their children do not show unique regulation activities when sharing with their parents. In other words, children who share with their parents show the same arousal behaviors as peers sharing with peers, evidenced by more significant changes in pupil diameters. To understand these differential patterns, it is essential to understand the differential roles that parents and children maintain. Self-expansion theories suggest that we tend to include close others in our own self-concept (Aron and Aron, 1996; Ketay et al., 2020). This is especially true for parents regarding their children (Thai et al., 2019; van Houtum et al., 2021), as parents need to care for, share resources, and empathize with their children (Bell and Richard, 2000) and model appropriate behavior (Davis et al., 2018). Alternatively, the child’s tendency to include their parent in their self-concept is less dominant, as they are not expected to care for their parent, and they are expected to seek autonomy (Thai and Lockwood, 2015; Thai et al., 2019). Given these differences in parent-child perspectives, it makes sense that parents will invest more resources when sharing with their children than with children sharing with their parents. In the current research, this investment can be seen via lower arousal represented by lower pupil diameter change.

The parent’s unique regulated behavior pattern may also be understood as an extension of previous research regarding modulated behaviors supporting their child’s needs. Some examples of this modulation include infant-directed speech patterns (“motherese”; Shruti et al., 2018) or modulated motoric responses (“motionese” Brand et al., 2002). Similarly, the current research suggests that parents still modulate their arousal response in older childhood, evidenced by lessened pupil dilation change than the other interacting groups, possibly to accommodate their child’s needs. Previous research describes stress contagion, in which parents pass their stress responses on to their children (Waters et al., 2014; Burkhouse et al., 2015). Therefore, it may be that parents naturally modulate their arousal responses, even in neutral settings, to model a calm environment for their child. This pattern of lessened pupil diameter change noted in parents indicates low arousal levels and low effort involved (Kinner et al., 2017). That is, parents seem to naturally remain calm, suggesting a subconscious behavioral pattern that parents enact with minimal effort.

Theoretical models suggest that parental prefrontal inhibitory circuitry influences their child’s emotion regulation neural networks such that parental behaviors effectively regulate their child’s behaviors (Hofer, 1995; Kerr et al., 2019). Importantly current findings extend research that focuses mainly on infants and young children to older children, suggesting that parents continue to modulate their emotional behavior as their children grow. We see this in day-to-day life when parents often try to downplay their fears, anger, or other strong emotions to modulate their response’s effect on their child. In fact, dynamic aspects of the parent-child dyad contribute to socio-emotional outcomes in young children (Lunkenheimer et al., 2020), and parent’s abilities and likelihood to adjust based on the context of the situation may be especially adaptive measure.

The current research extends the body of work concerning parental regulation of children’s stress and anxieties to the exploration of neutral, non-emotionally valanced experiences. Past research focuses mainly on parent-child dynamics and their influence on regulation during challenging or stress-inducing tasks (Miller et al., 2013; Perry et al., 2013; Paret et al., 2015; Armstrong-Carter et al., 2021). These studies contribute greatly to our understanding of how parents model arousal regulation and provide support for the critical role of the parent in teaching a child how to navigate emotion regulation in fear, stress, or anger-inducing scenarios; however, they tell us little about everyday scenarios that are not overly challenging. The current study provides insight into the neuro-behavioral responses in a neutral shared experience that is not especially difficult beyond its social component. Findings support the notion that parents continue to model arousal regulation behaviors in support of their child not only in stressful events but also during neutral interactive tasks with their child, such as sharing experiences.

A review of the literature on the parental brain suggests a parental caregiving network, consisting of the amygdala, hypothalamus, and dopaminergic reward circuitry activation that has been observed in response to infant cues (Feldman, 2015). Imaging studies highlight post-partum neural plasticity in the maternal brain (Kim et al., 2016) and activation of caregiving networks specific to paternal as compared with non-parent male brains (Diaz-Rojas et al., 2021, 2023). This would suggest that parents can adjust better than other populations. On the other hand, meta-analytic findings highlight differentiation in the neural response to one’s own vs. other children, with increased left hemisphere activation in response to one’s own child (Rigo et al., 2019). These findings suggest a more specific effect concerning reactivity to one’s own child rather than a general effect. Given the current literature, it seems that while general changes to the parental brain may plausibly affect a range of interactions, the effects are likely more pronounced when interacting with one’s own child.

### 4.1. Limitations and future research

Current data shed light on parent vs. non-parent responses, though findings are limited in their ability to pinpoint whether the effect is specific to parents sharing with their child or whether alternations in the parental neural networks lead to a more general effect. Future research may explore this question by comparing between two identical tasks in shared and individual conditions. Further, given the nature of the task and the low cognitive demand, theory supports the notion that parental pupil diameter differences noted in the current findings are most likely to reflect an arousal regulation process, though other considerations, such as cognitive load, cannot be completely ruled out.

## 5. Conclusion

The population of typically developing participants provides an archetype representing the neural-regulatory dynamics that can be expected when parents share with their children and vice versa. These findings have implications for our understanding of the neuroscience of parenting, suggesting that parents, even of older children and adolescents, tend to regulate their response when interacting with their child, a response that proves to be unique compared to others during shared experiences. Taking this dynamic into account, and with the support of future research in pathological cohorts, findings may direct future parent-led intervention methods aimed at replicating parental adapted arousal regulation, as captured in the current study, to improve the child’s socio-emotional development.

## Data availability statement

The raw data supporting the conclusions of this article will be made available by the authors, without undue reservation.

## Ethics statement

The studies involving human participants were reviewed and approved by The Department of Psychology Ethics Committee Bar-Ilan University. Written informed consent to participate in this study was provided by the participants’ legal guardian/next of kin.

## Author contributions

JY: primary writing, data collection, conception of study design, and data analysis. RG: conception of study design, data interpretation summary, and editing. MS: data collection and conception. OL: data analysis and interpretation. All authors contributed to the article and approved the submitted version.
